# Predisposition factors and control strategies of avian pathogenic *Escherichia coli* in laying hens

**DOI:** 10.3389/fvets.2024.1474549

**Published:** 2024-11-04

**Authors:** Paul K. Waliaula, Elijah G. Kiarie, Moussa S. Diarra

**Affiliations:** ^1^Department of Animal Biosciences, University of Guelph, Guelph, ON, Canada; ^2^Guelph Research and Development Center, Agriculture and Agri-Food Canada, Guelph, ON, Canada

**Keywords:** avian pathogenic *Escherichia coli*, colibacillosis, antibiotics, feed additives, pullets, layers

## Abstract

Shift in laying hens housing from conventional cage-based systems to alternatives has impacted their health and performance. Microorganisms colonize young chick in the early stages of their physiological and immune development. These colonizing microbes originate from parent and the environment. *Escherichia coli* is among the normal gut colonizing bacteria however, some *E. coli* strains known as avian pathogenic *E. coli* (APEC), cause local or systemic infections (colibacillosis) responsible of significant economic losses to the poultry industry. Potential APEC strains and other poultry gut microbiota are influenced by several factors such as housing system, and the use of feed additives (prebiotics, probiotics, symbiotic, among others). This review will discuss the status of pullets and layers immunity, gut health, and predisposing factors of colibacillosis. Dietary interventions and some colibacillosis mitigation strategies in pullets and laying hens are reviewed and discussed. With the development of sequencing technologies and the use of feed additives as alternatives to antibiotics, future studies need to understand some of the complex associations between the feed additives, the rearing environment, and their selective pressure on gut microbiota, including *E. coli*, and their impacts on immune development in pullets and hens.

## Introduction

The egg industry is one of the major sectors in the poultry value chain that attracts investment globally, and several countries have regarded eggs as the cheapest and healthiest source of animal protein (1, 2). Pullet rearing plays an essential role in the egg supply chain. Growers are mandated to raise healthy pullets to set a firm foundation for egg production that meets consumer’s demand (1). A major challenge encountered during pullet rearing is early mortality caused mainly by APEC infections, which interferes with flock uniformity, sexual maturity and performance (3, 4). Moreover, the poultry industry faces constant pressure to generate high-quality protein under different challenges, such as economic recession, animal welfare, and food safety (pathogens and antimicrobial resistance) (5). Conventional housing systems are still used globally in hens rearing despite being phased out in Europe since 2012 (6, 7). In Canada, more than 1,200 egg farms are raising about 24 million laying birds in conventional (battery) cages (57%) that produce 9 billion eggs annually (8). Raising hens in conventional low battery cages does not offer adequate space for the birds to behave naturally, which is a fundamental requirement according to the new legislations in some countries (9, 10). Thus, egg farmers of Canada are expected to phase out this cage system while establishing new Code of Practices and guidelines for pullet rearing (10–12). The adoptions of furnished and non-furnished cage systems decreased diseases prevalence and mortality rates (12).

Studies in Sweden between 2001 and 2004 on causes of mortality in laying hens revealed that colibacillosis was prevalent in layers (between the start of lay and 30 weeks) reared in litter-based housing system (38.7%) and conventional battery cages (38.6%) (10). Efforts have been deployed to phase out conventional low-battery cages and replace them with furnished and non-furnished cages systems to reduce mortality and improve performance. However, there is a need to adopt nutritional strategies, including the use of feed additives (pre-and pro-biotics, symbiotics, and phytobiotics) that have shown promising results in enhancing immunity and decreasing pathogens in the gut, especially APEC in laying hens. The aim of this review is to discuss the pullets/layers’ immune development, gut health, pathogenesis, and predisposing factors to colibacillosis. How dietary interventions could help in bolstering the immune system and mitigate infection in pullets and laying hens to improve productivity are also discussed.

## Avian pathogenic *Escherichia coli*

*Escherichia coli* (*E. coli*) is a commensal bacterium of the gastrointestinal tract (GIT), the pharynx and trachea of birds, animals and human (13). However, some *E. coli* strains are known to cause serious diseases such as cystitis, colibacillosis in birds and animals, pyelonephritis, meningitis/sepsis, and gastroenteritis in humans due to their possession and expression of various virulence factors (14). Extraintestinal Pathogenic *E. coli* (ExPEC) strains which causes diseases outside the GIT, are epidemiologically and phylogenetically distinct from intestinal pathogenic *E. coli* (15). These ExPEC pathotypes, including uropathogenic pathogenic *E. coli* (UPEC), Sepsis-Associated Pathogenic *E. coli* (SEPEC), Neonatal Meningitis *E. coli* (NMEC) and Avian Pathogenic *E. coli* (APEC) pose considerable threats to human and animal health (16, 17). The APEC isolates harbor more virulence genes including adhesins (*fimH, focG, hra, iha, kii, papA, papC, papEFG, sfa, sfaS*), toxins (*cdts, EAST11, pic, tsh, vat*), protectins (*iss, kpsMT K1, kpsMT KII, kpsMT K5, traT*), iron regulated systems (*ireA, iroN, iutA, fyuA*) and miscellaneous genes having various functions [*clbB, clbN, cvaC, H7 fliC, hemF, ibe10, ompT, malX (PAI), uidA, usp*] than the commensal *E. coli* strains (18, 19).

Colibacillosis is characterized by respiratory, systemic, and reproductive tract infections caused by APEC (16). It can affect poultry of all ages, leading to significant economic losses and compromising animal welfare in poultry production (20, 21). Colibacillosis requires predisposing factors such as infections by *Mycoplasma gallisepticum*, compromised mucosal barriers, immunosuppression related to stressors (vaccinations and viral infections), poor hygiene and ventilation in hatchery and poultry barns (21). Newly hatched chicks and pullets have weak and underdeveloped immune systems, making them more susceptible to APEC infections. This results in early mortalities of birds and decreased egg production in laying hens due to salpingitis-peritonitis syndrome (9).

## Transmission and pathogenesis of APEC

**Figure 1:**
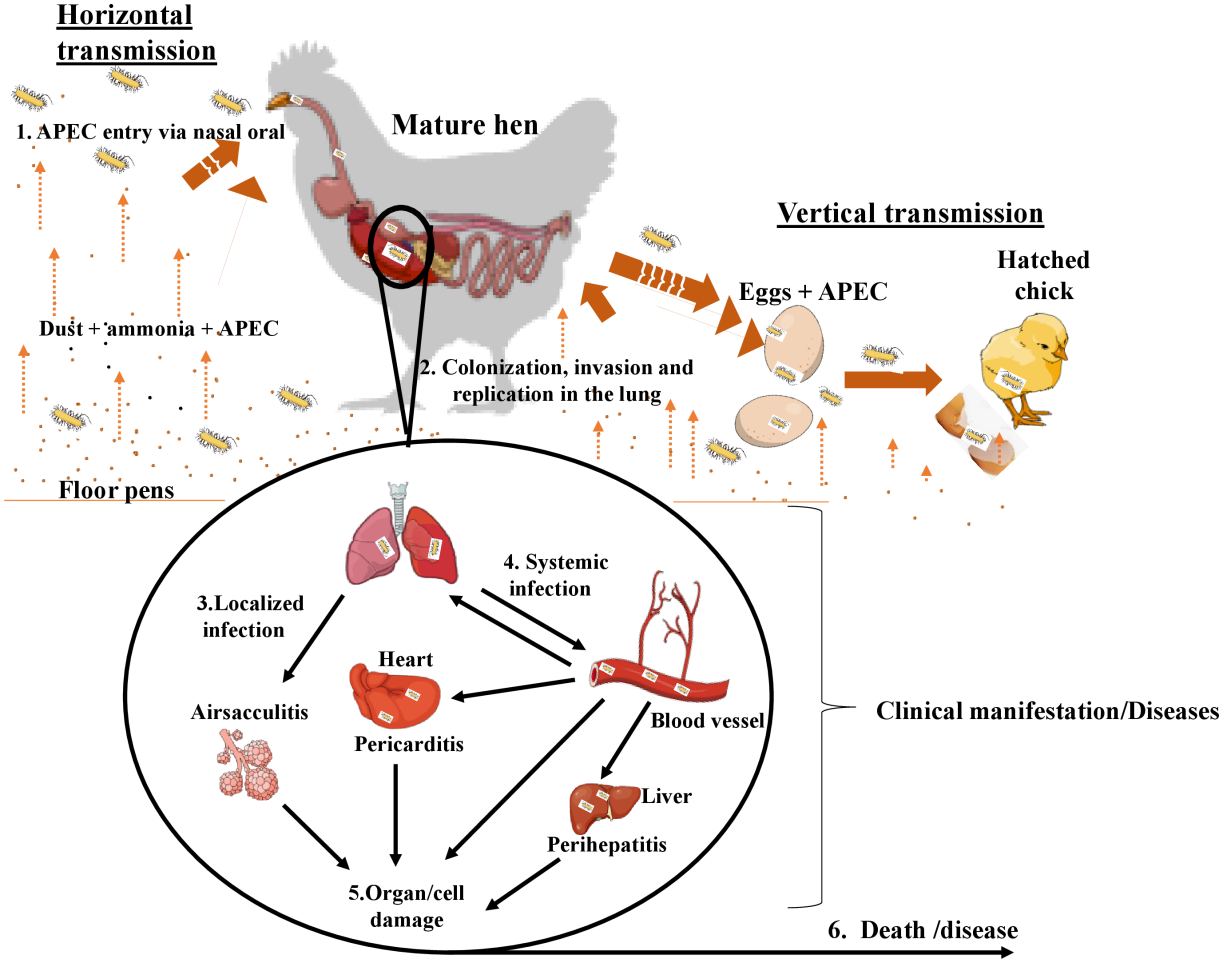
Transmission and pathogenesis of APEC infection.

The main transmission route of APEC in pullets and laying hens is via the fecal-oral route through inhaling contaminated dust (horizontal transmission) in hatcheries or production houses (22). The vertical transmission route is hypothesized to emanate from the infected reproductive tract (22–24). However, the pathogenesis of APEC and how it causes yolk sac infection are unclear. Investigations indicated that the APEC mortality rate ranges between 10 and 20% in 48 h after hatch, primarily due to septicemia (25). Pathological signs can include lung congestion, splenitis, and edematous serous membranes. A couple of days after the infection, fibrin-heterophilic polyserositis appears within the pericardium, air sacs, pleura, and perihepatic tissues. The incubation period ranges between three to five days after the infection (Figure 1). Previous studies showed a close association between the first-week mortality and the breeder hen age; the older the breeder, the higher the risk of first-week chicks’ mortalities (16). Despite understanding their source and transmission route, the mechanism by which APEC establishes infections needs to be clarified, and experimental findings disclose substantial inconsistencies (26, 27). Studies indicated that APEC can colonize the chicken gastrointestinal and respiratory tracts without causing diseases and only their translocation to extra-intestinal sites in the presence of production-related stress induced the disease (Table 1). As shown, *E. coli* isolates from colibacillosis harbor virulence genes that encode adhesins, invasins, iron acquisition systems, toxins and protectins, which play an important role in its pathogenesis (16). Adhesion to the host cells is an important stage of APEC pathogenesis and is mediated by various adhesins (Table 1). Type 1 fimbriae play a crucial role in the adherence of APEC on the epithelial cells of the respiratory tract during the initial stages of the infection (14, 16, 28–30). In contrast, the expression of P fimbriae and S fimbriae contributes later to APEC pathogenesis. Anti-type 1 fimbriae serum and D-mannose, cellular receptors for type 1 fimbriae, could block chicken tracheal colonization by certain APEC strains. Curli fimbria is associated with bacterial biofilm formation and cell invasion (31). Temperature-sensitive hemagglutinin (*tsh*) mediates colonization during the first stages of respiratory tract infections (32). Invasins virulence genes facilitate the entry of APEC into host cells (32–34). These virulence-associated factors facilitate APEC adhesion, host cell invasions, evasions from phagocytic cells, colonization, multiplications/proliferation, cell lysis and damage, as well as systemic dissemination of APEC infections, and eventually colibacillosis infection in chicken (Table 1).

**Table 1:** The roles of virulence associated factors/gene in pathogenesis of colibacillosis/APEC infections.

Virulence factors	Designation	Cluster/Genes name	Functions/role in pathogenesis	Location	Year of publication	Ref.
Adhesins	Type 1 fimbriae	*fimC* and *fimH*	Colonization, biofilm formations by binding receptor D-mannose	Chromosomes	2017, 2020, 2020, 2014	(14, 19, 28–30)
	P fimbriae	*papA papC, papF, papG* genes	Colonization and stimulation of cytokine production	Chromosome	2017, 2000	(79, 91)
	Stg fimbriae	*stg*	Colonization/attachment	Chromosome	2021	(193)
	Curli fimbriae	*cfa*	Colonization, biofilm formation & activation of immune system	Trachea and intestine	2022	(31)
	Autotransporters	*aatA/aatB*	Colonization	CoIV plasmid	2011, 2017	(94, 95)
	Temperature sensitive Hemagglutinin (*tsh*)	*tsh*	Colonization, adhesion, biofilm formation and cytopathic effects	CoIV plasmid	2019	(32)
Invasins	Invasion proteins	*ibeA, ibeB, tia* and *gimB*	Biofilm formations, Resistance to oxidative stress, invasion, biofilm formation, colonization and proliferation	Chromosome	2011, 2012, 2021	(33, 34, 194)
		*ibeR*	Invasion, resistance to environmental stress, serum resistance and virulent gene expression	Chromosome	2021, 2015	(34, 195)
		*ych O*	Biofilm formation, motility, invasion, adhesion, colonization and gene expression	Chromosome	2016	(196)
Toxins	Putative avian haemolysin	*hlyA, hlyE, hlyF*	Cell lysis and damage, Pore forming toxins Colonization	Chromosome/CoIV plasmid	2021, 2000, 2016	(34, 197, 198)
	Vacuolating autotransporter toxins	*vat*	Cytotoxic effect, Biofilm formation, motility, virulent gene expression, Agglutination	Chromosome	2015	(199)
	Cytolethal distending factor	*cdtB, cdtC*	Cytotoxic effect, Biofilm formation, Motility, colonization	Chromosome /Plasmid	2015, 2019	(200)
	Heat-stable enterotoxin (EAST-1)	*astA*	Adhesion, induction of vacuolization, biofilm formation, colonization	CoIV/CoIBM plasmid	2019	(201)
	Shiga toxin variant	*stx2f*	Biofilm formation, adhesion, colonization	Chromosome /Plasmid	2022	(202)
Protectin/serum survival	Transfer protein	*traT*	Inhibitions of classical pathways/complement resistance	IncF plasmid	2021, 1985	(34, 203)
	Capsular antigens	*kpsMT2,* *kpsMT3, iss*	Intracellular survival, colonization, proliferation, adhesion, serum resistance	Chromosomes	2021, 2016	(34, 204)
	Outer membrane protein	*ompT*	Immune system evasion, intracellular survival	Chromosome, CoIV plasmid	2005	(205)
	Lipopolysaccharides (LPS)	*wzy, waa lpX*	Intracellular survival, adhesion, invasion	Chromosome /ColV plasmid	2014, 2019	(206, 207)
	CoIV, CvaC	CoIV, cvaC	Colonization	ColV plasmid	2014	(34, 208)
Iron acquisition system	Aerobactin	*iucA/AuC/iucABCD*	Iron acquisition, siderophore	CoIV plasmid	2022, 2013	(31, 209)
	System “*sit”* genes	*sitABCD*	Transportation of Fe, Mn	CoIV plasmid/IncF/IncN plasmid	2023	(210)
	Salmonechelin	*iroBCDE* and *iroN*	Fe utilization, siderophore receptor	CoIV plasmid	2015, 2012	(1, 116)
	Heme receptor	*chuA*	Transportation of *chuA* protein and Heme utilization	Chromosomes	2023, 2012	(114, 116)
Miscellaneous	Phosphate transport system	*pst B*	Colonization, resistance to bactericidal activities and oxidative stress	Chromosomes	2015, 2000	(13, 211)
	Two VI secretion systems	*hcp, clpV*	Adherence, invasion, biofilm formation		2010	(212)
	Transcriptional regulator	*yjjQ, rcsB, ntrC*	Colonization, Biofilm formation, motility,		2015, 2022	(13, 213)
	Yad fimbriae Yqi fimbriae	*Yad, Yqi*	Colonization and biofilm formation	Chromosome	2022, 2016	(214, 215)
	Secreted autotransporter toxin	*Sat*	Cytotoxic effect, colonization, biofilm formation, motility Impairment of tight junction		2022	(216)
	Serine protease autotransporter	*Pic*	Agglutination, biofilm formation Adhesion, colonization, evasion Serum resistant		2019	(217)
	Serine protease	*espC*	Biofilm formation, adhesion, cytopathic effect		2004	(218)

## Pullet and layers immune system development

The development of birds’ defense mechanisms starts during the embryonic stage, with eggshells offering physical barriers against damage and infections. Apart from the eggshell, the four main parts of an egg’s inner structure (the yolk, vitelline membrane, and egg white) offers protections to the developing embryo. Throughout the embryo’s 21-day development, they supply energy and nutrients as well as chemical and molecular defenses against microbial invasion. Embryonic development (ED) takes 21 days and the first sign of developing immune system is observed at day 10 (ED10) and at ED18, chicken embryo is immunocompetent and able to produce both innate and adaptive immune response to pathogens (35). Innate immune system of chicken embryo starts to develop in early embryonic stage (ED2), with key immune cells (CD2+ AND CD45+ cells) present in in yolk sac, before liver and thymus are fully developed (36). Granulopoiesis begins early in embryonic development with the expansion of the granulocytic lineage (between ED7 to ED20) in the yolk and splenic primordium and granulocyte differentiation in the liver at ED15 (35). Chicken embryos are unable to mount adequate immune response until ED12. In chicks, analysis of heterophil functionality allows to describe their activities, such as phagocytic activities, degranulation, microbial killings, and oxidative burst, which appears to decrease in mature birds compared to newly hatched chicks. At ED18, the chicken embryo is stimulated with sufficient heterophils that enhance immune functions suggesting that they are already functional at that time (35). However, uncertainties exist about when heterophils begin to be fully functional.

Chicken embryo is immunocompetent and able to produce both innate and adaptive immune responses to pathogens. However, as the embryo develops, innate defenses in the egg disappear, allowing the growth of pathogenic bacteria however, this needs to be fully characterized. Moreover, immunocompetence appears a few days post-hatch, and during this period, the chicks rely on maternal antibodies for protection against microbial infections (20, 21). Innate immunity is non-specific but, it offers the first line of defense against a wide range of pathogens in birds and does not confer long-lasting immunity. The components of the innate immune system are physical and chemical barriers, including blood proteins and cellular components. The innate immune system reacts promptly against invading pathogens by quickly instructing antigen-presenting cells (APC) to activate and secrete cytokines that regulate T and B cells to mount suitable adaptive immune responses. Activation of APCs in response to pathogens is mediated by pattern recognition receptors (PRR) resulting in the production of pro-inflammatory cytokine and co-stimulatory molecules, which are involved in the activation of adaptive immune response (37).

Maternal antibodies and innate immune cells such as macrophages and monocytes, play important roles in the chicks’ defenses against pathogens since adaptive immune functions have not yet been fully developed during the first three weeks of the chicks life, which rely solely on maternal antibodies. The maternal antibodies reduce gradually as chicks age resulting in an increased susceptibility to disease (9). Thus, early interventions such as nutrition to promote the development and maturity of the immune system of chicks are needed. The development and maturity of the immune system in laying hens takes about eight weeks after hatch, and this underdeveloped immune system in young chicks increases the susceptibility to yolk sac infections (38, 39). Studies using ileum and jejunum in laying hens revealed that IgM and IgY gene expressions peak at weeks one and five of age respectively, while IgA expressions increased with the hen’s age during the growing period (40). At the week eight, the sexually immature hens undergo bone and reproductive development until about 17 weeks of age. Contrary to the laying phase, which is characterized by decreased cytotoxic T lymphocyte (CTL) and γδ T cell levels and increased innate immunity (38, 40). During the onset of lay and at the egg production peak, the estrogen and corticosterone hormone levels rise along with a remarkable change in the immune cells proportion and a reduction in cellular response, leading to an increased susceptibility to pathogenic bacteria (*E. coli* and *Salmonella*) (38). Currently, limited studies exist on immune parameters during the pullets, and laying phase.

## Immune system and the predisposition factors to APEC

### Hatchery hygiene

**Figure 2:**
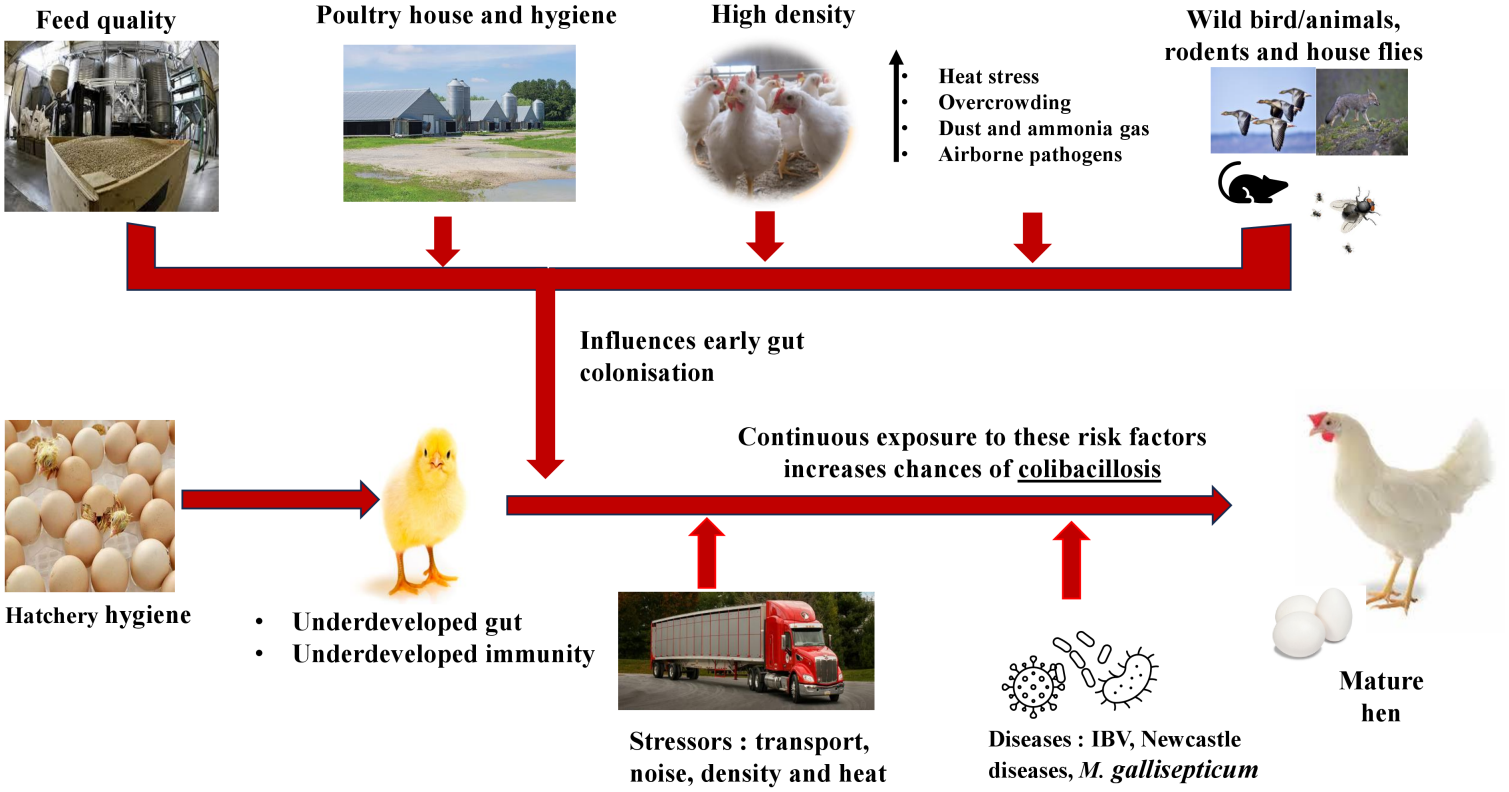
Predisposing factors to colibacillosis infections in pullets and laying hens.

The hatcheries occupy a central position between breeder and producers thus, optimized hatchery hygiene plays an essential role in pathogen prevention across the production chain (41). The first week is crucial in transforming chick’s life and its overall performance (42). From the hatcheries to the brooders’ house, the chicks grow and adapt to new feeds, and environmental changes while fighting against diseases. The large-scale production in commercial hatchery and transportation of chicks over to brooder house, predisposes them to infections especially by pathogenic *E. coli* (42). During the first week of life both layers and broilers are highly susceptible to infections caused by APEC or *Enterococcus faecalis,* which accounts for more than 50% mortalities during this period (4). These infections are thought to originate from hatcheries and parent flocks. Kemmett et al. (43) has demonstrated that intestinal tract of day-old broilers chick can be colonized by APEC carrying at least 10 virulence-associated genes. Breeder hens raised on floor/litter get the eggshell and eggs contaminated with bacteria such as *E. coli* which could infect the chicks during the incubation (44). While in the hatchery, the chicks infected via the vertical route succumb to death from yolk sac infection (omphalitis) (16, 42). Thus, it is important to maintain a pathogen-free hatching environment to prevent contamination and the spread of pathogens in poultry production chain. Figure 2 shows potential sources of infections including the hatchery and transportation however, improper cleaning, disinfection and people handling the chicks could result in infection as well.

### Poultry houses

The initial exposure to APEC could happen in the hatchery from the contaminated/infected eggs (4, 25). Systemic infection usually needs predisposing factors (infectious/non-infectious) such as nutritional deficiencies, toxins, other diseases, and the under-developed immune system of the bird. Such stressors are experienced mostly in commercial set-ups, and they increase corticosterone levels and susceptibilities to colibacillosis. A high prevalence of bacterial, viral, and parasitic infections could occur in litter-based and free-range systems (45). In commercial farms, layer hens are reared in a cage system, classified into conventional and enriched cages or an alternative housing system (46). Conventional cages have limited spaces for bird’s movement while lacking nests and perches to allow the expression of natural behavior (18, 30). The alternative housing system includes indoor (single-tier and multi-tier), and outdoor (free range and organic) housing systems designed to meet bird’s comfort (10). More research is needed to ascertain effects of these housing designs and stocking density on colibacillosis in pullets and laying hens.

The Canadian Code of Practice for the Care and Handling Pullets and Layer, established in 2017, offers the detailed guidelines on housing design and stocking density of laying hens (11). High-stocking density generate dust and ammonia in these housing systems (10), which causes health problems for birds (47). The increase in ammonia levels in poultry houses damages the respiratory system of the birds, predisposing them to infectious agents such as bronchitis virus (IBV), Newcastle disease virus and *Mycoplasma gallisepticum*, which may play a role in the APEC pathogenesis (47). Therefore, unfavorable housing environments that generate a lot of dust and ammonia result in the inhalation of large quantities of contaminants which are not completely cleared in the respiratory system making birds highly susceptible to colibacillosis (48). Inhalable dust concentration varies with housing system: aviary ranges (1.3–9.5 mg/m^3^), conventional cages (0.2–2.3 mg/m^3^), and enriched system (0.4–3.5 mg/m^3^) (49). Bacterial counts ranged between 10,000 – 8,000,000 cfu/m^3^ in different housing systems, and indoor *E. coli* counts have been reported to reach to1,000 cfu/m^3^ with slightly a higher concentration in the aviary (50). In the dust, *E. coli* can survive for extended period, and its airborne transmissions can result in its spread within and between poultry houses (26, 50).

Dust generation and ammonia emissions above 70 ppm can reduce spleen weight, lysozyme and globulin concentration while limiting lymphocyte proliferation (51). High ammonia level of more than 25 ppm in laying houses has been reported to decrease feed intake and growth rate (35). The appearance of a NH_3_ concentration of 15 ppm affected the trachea microbiota of chicken and increased the possibility of upper respiratory tract infections. In broilers, NH_3_ increased *E. coli* and *Shigella* numbers in lung tissue and activated inflammation (52). Moreover, high concentrations of NH_3_ over a long time resulted in respiratory illness, increased susceptibility to APEC infection, affected egg quality and reduced egg production (51). An investigation of Hy-Line Brown laying hens showed that high NH_3_ and temperature jointly increased IgG and decreased IgA (53). Generally, NH_3_, respirable dust and bacteria are much higher in the aviary and floor housing system than in conventional cages, with the least in furnished cages (54).

### Bird stocking density

Stocking density refers to the number of hens or hens’ body weights per unit area, and space allowance is the space provided for each hen (55). Providing adequate space relative to a hen’s body area would increase freedom of movement and allow it to adequately perform all behavior in their repertoire (56). The current recommended and approved stocking density by the National Farm Animal Care Council (NFACC) in Canada for poultry rearing ranges between 284 cm^2^/bird for high density and 854 cm^2^/bird for low density (11). Spacing allowances, types of housing systems, and their effect on performance of laying hens are presented on (Table 2). These factors influence birds’ welfare while increasing stress level and diseases susceptibilities (57). A study on impact of cage density revealed that there is an increased risk of mortality in high-stocked density barns, but the causes of mortalities were not established (56). The findings indicated that the housing system could influence bird’s performance and health (58). Furthermore, high stocking density was negatively correlated with the development of immune organs (59).

**Table 2:** Spacing allowance, types of housing system and their effects on performance in laying hens.

Spacing allowance (cm^2^/bird)	Age at the start and end of the trial in weeks	Duration of study in weeks	Breed/genetic of birds	Type of housing system	Impacts of housing system	Publication years	Reference
600	17–78	61 (sampled at 28 and 62)	Hy-line white and Hy-line Brown	Conventional and furnished cages	Egg produced in furnished cages have higher bacterial shell contamination than conventional cage Age has no significant difference on *Enterococcus* count between week 28 and 62 Number of aerobic microbes was higher at 28 weeks, while proportion *Enterobacteriaceae* from eggshell was higher at 62 weeks than 28 weeks	2008	(46)
CC: 200 (for chicks) until (5wk); 400 (from 5–18 weeks) 688 Floor pen: 6,115–6,990 for 21–24 birds in each pen	Day 0–50	50	Lohmann Brown, Lohmann white H & N white Cross breed between Rhode Island male and Barred Plymouth Rock female	Floor pen Conventional cage	Yolk color was greater for eggs laid on floor than those in cages. Lower albumen height in eggs from the floor tan in cages due ammonia. Eggs from cages had lower coliform and *E. coli* contamination than those the floor and nest-boxes *E. coli* contamination was higher for LB than LB eggs	2009	(47)
CC: 640 Furnished cages: 750	17–65	48 (sampled at 8 interval)	Brown layers	Conventional cages Furnished cages	There was no significant difference between conventional and furnished cages for either gram negative flora or total aerobic flora Egg shell contamination with total aerobic/Gram-negative flora in furnished and conventional cages was not influenced by nest floor material (wire or artificial turf)	2005	(48)
CC: 77 EC: 1505	35 to 85	50 (sampled at 32–51 and 52–85)	Hy-line brown White leghorn (W-36)	Conventional cages 3-tiers Enriched cages: 2-tiers Free range	Hen day egg production was higher in FR and CC than EC, Hy-line hens reared in EC had the lowest hen day egg production than other treatment. White leghorn had lowest feed intake and highest feed conversion ratio in EC than CC and FR FR performed better for egg quality than CC and EC, and Hy-line-Brown had better egg quality than white leghorn FR hens had higher cloacal bacterial contamination (aerobes) (anaerobes and coliforms) than CC and EC	2022	(49)
EC 750 FR (9 hens per m^2^)	26–51	25 weeks (sampled on 21 and 51)	Isa brown	Enriched cages Free range	Heaviest eggs (63.3 g) were laid by hens raised in free range at 51 wks of age and lightest eggs were found in same housing at 26 wks of age (58.0 g) Heaviest eggs in EC were from hen aged 51 wks of age (61.7 g) FR eggs contained more lysozymes than EC eggs	2019	(50)
EC: 750	16–38 weeks	20 (sampled at 18 and 38)	Super Nick white egg layers (pullets)	Enriched cage	Feeding layer hen with 1 kg lignocellulose (LS) improved Highest mean of egg production and egg weight (81.8% and 57.3 g). Total aerobic bacterial load of eggshell was lowest in 1 kg LS group. Laying hens fed with 0.5 and 1 kg LS had longer villus height	2020	(219)
CC: 550 FR: 10 m^2^/bird in poultry house, and 4 m^2^/bird on the range	25–75 weeks	50 (sampled at 25, 35, 45, 55, 65 and 75)	Hy-line hen	Conventional cages Free range	Eggshell quality, egg weight and shell thickness increase as the flock age and were higher in CC than FR Total bacterial and *Enterobacteriaceae* was relatively low in CC eggs and higher in FR eggs	2014	(52)
Low stocking density (13 birds/m^2^) High stocking density (23 birds/m^2^) Same density (2.4 birds/m^2^)	7–17 weeks Same density (18-28wks)	10	Lohmann leghorn selected lite	Floor pen	At the end of pullet rearing phase, pullets under high stocking density had lower number of T lymphocytes γδ T in blood, spleen and ceca tonsils showed a higher heterophil to lymphocytes ratio Stocking density during rearing phase had short-and long-term effect on the immune system of layer pullets	2021	(53)
CC: 492 EC: 780 AV: 1120	23–32	10 (weekly)	Lohmann LSL lite	Conventional cages Enriched cages Aviary	Laying rate and egg weight were similar in EC and CC (96.5% and 59.5 g; *p* > 0.05), while AV had lower rate (77.2% and 58.6 g) (*p* < 0.001) Almost 70% eggs in EC are laid in the nest, whereas In AV almost all eggs are laid on litter. Rate of clean eggs was about 77% for EC and CC compared to 14% in AV	2020	(54)
Cage system: (H) 450, (I) 675 (L) 900	11	20 (sampled at 24th and 43rd)	Langya hens	Cage	Hen from high stocking density had lowest body weight, egg weight and yolk color score index Increased stocking density is associated with bacteria taxa with threatening health problem.	2019	(38)

AV, aviary; EC, enriched cages; CC, conventional cages; FR, Free range.

Infectious diseases are a significant concern for hens raised in high stocking density (57). Gast and his colleagues (60) observed higher *Salmonella* Enteritidis counts in the liver and ovaries of experimentally infected hen housed in a high stocking density enriched cage system (648 cm^2^ per bird) than in hens housed at lower (973 cm^2^ per bird) stocking density (60). High stocking density (284 cm^2^/bird) induces physiological stress by stimulating the production of corticosterone (CORT) hormones from the hypothalamic pituitary adrenal axis in response to appetitive and aversive stimuli, which act upon behavior, metabolism and immunity (51). Eugen et al. (61) reported high anxiety, blood pressure, and CORT hormones in pullets, when the stocking density (SD) increased from low SD (500 to 1,429 cm^2^/chick) to high SD/overcrowding (56 to 167 cm^2^/chick) during the first 10 weeks of age in conventional housing system. Overcrowded barns were found to increase the *Salmonella* Enteriditis population in chicken caeca, which might contribute to food safety concerns (62). Stress and unconducive housing conditions are understood to predispose both young birds and adult birds to colibacillosis (63). As summarized in Table 2, the adoption and use of enriched cages might help improve performance and immunity of layer hens.

### Stressors

Stress is an adaptive response to threats on animal homeostasis; changes in immune status and response reflect animals’ reaction to stress (64). Poultry are confronted with a wide range of acute and chronic stressors in their housing environment that might threaten their welfare and health by modulating their immune system (65). The parameters used to measure these stressors are heterophil and lymphocyte ratio, corticosterone, glucose, and catecholamine levels (66). Studies have demonstrated that immunological stress induced by *E. coli* endotoxin lipopolysaccharide (LPS) causes pullets sickness, including high fever, reduced feed intake, and changes in birds’ behaviors (67). Nutritional factors, including high levels of dietary polyunsaturated fatty acids (PUFA), deficiencies of vitamins (A E), selenium, zinc, and manganese, and the presence of mycotoxins, and other toxic substances, are the most important stressors during growth and development. Reproductive stages, and dietary changes (starter, grower, and finisher) also interfere with bird’s immune functions. For example, poor feed formulation with deficiency or excessive vitamin A increases chicks’ susceptibility to *E. coli* infections, followed by a decreased immune response (68). Additionally, birds can be affected by other stressors including environmental conditions (temperature, relative humidity, heat and cold stress), infectious agents (bacterial/viral), and transportation stressors.

The immune response in bird differs according to the period and severity of the stressor and its physiological and nutritional status (Figure 2). Birds are known to maintain relatively constant temperatures of their internal organs. The housing system for laying hens has been shown to influence the immune cells (basophils, lymphocytes, eosinophils, and phagocytes) functions against pathogenic bacteria (*Salmonella* spp.) due to intestinal damages and the increase of inflammatory cytokine concentrations (69). A high ambient temperature increases chickens’ energy requirement, resulting in a significant loss for a less efficient feed conversion to eggs or meat, which has a detrimental effect on health and performance (70). High relative humidity and ambient temperature induce heat stress (HS), affecting performance, welfare, and bird’s health (70). Similar to HS, cold stressors interfere with bird’s performance by decreasing body weight and egg production in laying hens, and all these factors contribute to increased susceptibility to infectious diseases (38). However, the mechanism by which it causes colibacillosis is unclear and needs more research.

Heat stress occurs when the heat generated in the body surpasses its dissipation capacity, and the body cannot get rid of excess heat, resulting in reduced feed intake, body weight, egg production, and increased susceptibility to infection (71). Heat stress also negatively affects intestinal development, particularly intestinal epithelium integrity. It destroys crypt depth and stripping, resulting in reduced villi height, and decreases epithelial cell area ratio, promoting growth of pathogenic bacteria such as *Salmonella* and *E. coli* (72). Very high temperature influences a bird’s immune responses by inhibiting lymphocyte functions (38). Heat stress is reported to increase intestinal permeability through the physiological adaptation process. It increases the peripheral blood flow and decreases intestinal blood supply, resulting in hypoxia and oxidative stress (73). Continuous exposure to cold stress results in increased corticosteroids that cause an imbalance of the bird’s metabolism, increasing the susceptibility of the birds to diseases, especially colibacillosis (74).

Naturally, living cells balance the formation and inactivation of reactive oxygen species (ROS) and reactive nitrogen species (RNS) from mitochondria. However, due to different stressors, the free radicals’ productions exceed the ability of the antioxidant system to neutralize them, resulting in oxidative stress that causes damage to unsaturated lipids in plasma membranes, leading to DNA damage and disruption of membranes and cell integrity. Membrane damages are linked with reduced efficient nutrient absorption, leading to decreased performance, immunity suppression, and increased susceptibility to infection (75). For instance, hens are characterized by weak immune systems during their first week of life and peak production phase (32–35 weeks of age) and are highly susceptible to pathological alterations caused by *E. coli*. At the peak production phase, *E. coli* can colonize the oviduct (salpingitis) when estrogen levels are high, along with a weakened immune system (76).

Poultry transportation is one of the technological stresses encountered in the commercial industry (77), and the size and age of the bird greatly influence tolerance to challenges experienced during handling and transportation (78). Transportation is a multifactorial and stressful process that subject poultry birds to a noisy environment, poor handling during loading and offloading in trucks, highly stocked into designated net cages, deprivation of food, water and long transportation hours where they are exposed to vibration, heat, noise and cold. Watts et al. (79), reported higher moisture and heat production in smaller birds than larger birds. Heat stress was confounded in birds stocked at high density during transportation, which change the stress parameters in birds’ body predisposing them to bacterial infection and mortality (79). Thermal stressors experienced during birds’ transport have the potential to severely reduce welfare, contributing greatly to stress, disrupting homeostasis that increases the susceptibility to infection (80). Improper disinfection of net cages used initially for flock transportation might harbor infectious agents such as APEC, which can be transmitted to birds. Moreover, frequent social encounters could exacerbate negative stress induced behaviors for example cannibalism and feather pecking in high stocking density barn, which predisposes the hens to colibacillosis (10, 11). Stress in chicken can have a profound long-lasting effect on behaviors especially when experienced at early life and pullet stage (81, 82). However, the stress levels and its consequences vary during different stages of egg production (82). There is need for research to understand the correlation between stressors and chicken health and egg production.

## Nutritional requirements and colibacillosis

Nutritional requirements for pullets and laying hens are complex and should be managed carefully to ensure optimal health, productivity, and robust immunity. The lifecycle of laying hen is divided into pre-and post-sexual maturity (83). During pre-sexual maturity (pullet phase), poultry nutritionist formulates feeds to build a firm foundation for future production by ensuring the least mortality, optimum health status, proper sexual maturity time, and flock uniformity (84). Diets for laying hens are formulated to optimize performance, prolonged peak laying period, and optimum immune functions. The pullet phase is the most rapidly growing and important period of the hen’s life, which is categorized into four stages by age: 0–6 weeks, 6–12 weeks, 12–18 weeks, and 18 weeks to the age of first lay (83). The initial post-hatch phase of the chick can utilize nutrients from the yolk sac for 72 h and thereafter rely on exogenous nutrients. During this period, the quality of feedstuffs is of great significance to pullet health as a strong correlation exists between body development and laying performance and its ability to extend lay up to 100 weeks of age (83).

Chicks are usually fed crumbles starter diets for the first 4 weeks of post-hatch, with a relatively high-energy content (12.3–1.4 MJ/kg) derived from carbohydrates and fats and with an increased calcium concentration (1.05–1.10%) to promote skeletal development and crude protein (18–20%) for rapid structural and immune development (85). Sometimes, starter diets can be prolonged to 6 weeks, depending on the chick’s weight. Certain feed additives such as fatty acid, probiotics, prebiotics, and symbiotics are used in starter diets to enhance gut barriers and immune response against pathogens such as *E. coli*. Grower diets are generally fed from four to 10 weeks of age (WOA) and have lower energy (11.9–12.0 MJ/kg) and calcium (0.9–1.10%) content than starter diets (85). As about 95% of the skeletal development occurs during the grower phase, an adequate amount of calcium and phosphorous needs to be supplied to prevent osteoporosis in mature hens (86). At 10 WOA, pullets are fed on diets that contain low energy density to minimize incidences of overweight and increase gut holding capacity by promoting gastrointestinal organ development (87). The onset of sexual maturity begins at ~17 WOA, and the commencement of egg production is determined by age, body weight, body fat content, and increased light intensity.

Nutritional stressors can play a significant role in predisposing pullets and hens to colibacillosis. Imbalances of nutrient intake, for example, vitamins (A and E), proteins, and minerals, can compromise the immune system of hens, making them susceptible to colibacillosis (68). Studies on the resistance to *E. coli* infection using chicks fed diet depleted (0 μg/kg), sufficient (0.85 μg/kg) or excess (1,000 mg/kg) in vitamin A (59), revealed that excess or insufficient vitamin A resulted in an increased susceptibility of chicks to *E. coli* infection accompanied by reduced immune response by impairing IgA and IgG production (68). A study on the effect of dietary vitamin E type (synthetic 22.00 mg), vitamin E level (natural 220 IU/kg) in broiler male chicken challenged with LPS showed that natural vitamin E had a significantly lower LPS-induced inflammatory response than synthetic vitamin E, this suggests a protective effect from vitamin E (natural type) in case of bacterial components (88). These studies indicate that vitamins A and E have anti-inflammatory responses that protect chicken health against bacterial infection. However, nutritional deficiencies caused by linolenic acid, iron, and selenium impair immune functioning and increase susceptibility to infectious diseases (89, 90). Thus, these nutrients are required during the acute or chronic stages of the disease. Certain nutritional compounds, such as fatty acids, vitamins (A, C, D, and E) have anti-inflammatory properties, whereas others, including probiotics, prebiotics, herbal and plant extracts, and long polyunsaturated fatty acids, have immunomodulatory features (88). Diets that contain inadequate levels of protein or amino acids can interfere with immune functions and increase susceptibility to microbial infections, especially in pullets and laying hens. Therefore, there is a need to identify nutritional strategies to enhance the immune system of chickens against microbial infection.

## Control measures against APEC

### Antibiotics

The most common approach for controlling bacterial infectious diseases, including colibacillosis, is the use of antibiotics (91). However, the use of antibiotics in animal production has been linked to an increased antimicrobial resistance (AMR) prevalence (91, 92). Despite the presence of APEC in the oviduct, 62% of hens would continue producing eggs (93, 94). Antimicrobial-resistant *E. coli* isolates from laying hens have been reported in Switzerland (94), Belgium, Germany, and Italy (95, 96). Isolates were resistant to sulfamethoxazole, nalidixic, tetracycline, trimethoprim, ciprofloxacin, and ampicillin (94, 95). The egg industry has made significant progress in reducing antimicrobial use (AMU) and transitioning toward more humane husbandry practices such as using furnished cages, cage-free production, and pasture-raised hens. A study by Aguinos (97) reported tetracycline and gentamycin resistant *Salmonella, Campylobacter*, and *E. coli* in laying hens. Based on this study, more surveillance data on AMU and AMR in the egg industry is warranted (hatchery, pullet, and laying phase). In the United States of America (USA) most antimicrobials administered in egg-laying hens are via the feed; ionophores (monensin and salinomycin) are used in the pullet phase, and bacitracin is utilized in both pullets and layers to control *Clostridium perfringens* infections, and chlortetracycline for treatment of *E. coli-*associated infections (98). In the United Kingdom, tetracycline, penicillin, macrolides, aminoglycosides, pleuromutilins, fluoroquinolones, polymyxins, and lincosamides are used in laying hens (99). In the European Union colistin, neomycin, tylosin, oxytetracycline, chlortetracycline, and erythromycin are approved for use in laying hens while, the recommended antimicrobial use in the Canadian poultry industry includes penicillin G, neomycin, chlortetracycline and oxytetracycline (97, 100).

### Tetracycline

The Tetracycline class of antibiotics includes chlortetracycline, oxytetracycline, and doxycycline, which inhibit bacterial protein synthesis (101). Chlortetracycline (CTC) is the most common antibiotic used in feeds for pullets and laying hens. According to a 2021 DANMAP report from Denmark, penicillin and tetracycline were the only two antibiotics used in layers (102). Chlortetracycline is the second in-feed antimicrobials approved in USA against *E. coli* associated infections during the laying period (98). In Spain, Moreno (97) showed that day-old chicks are regarded as the source of antimicrobial-resistant bacteria for laying hens, with tetracycline resistance (75%) being the most prevalent (103). In Ontario, tetracycline resistance in *E. coli* isolates in sentinel sites ranged from 26 to 69% in hens (97). Tetracycline-resistant *E. coli* isolated from poultry has a likelihood to become resistant to additional antibiotics (104). Moreover, the resistance might be conserved in bacterial populations over time, regardless of selection pressure, which might lead to an overall increase over time. Tetracycline resistance can be plasmid-mediated via horizontal gene transfer by mobile genetic element such as transposons, and integrons (104). The identified genes include those conferring resistance by efflux pumps such as *tet(A), tet(B), tet(C), tet(D),* and *tet(G)* (105).

Moreover, rearing layers in conventional battery cages at close proximities and high stocking density can heighten the spread of these resistance genes (105, 106). Studies have revealed that *tet(A)* and *tet(B)* are the most detected tetracycline resistance genes in commensal and pathogenic *E. coli* isolates from poultry, with *tet(A)* being the most prevalent (105). For example, a study in broiler chickens examining *E. coli* isolates from colibacillosis lesions identified a high prevalence of *tet(A)* and *tet(B)*, with 96.7 and 38.3%, respectively (105, 106). These two genes have also been found in chicken manure and soil samples around poultry farms, emphasizing the environmental dissemination of this resistance determinant (105, 106). The presence of tetracycline resistance genes in such environments highlights the importance of prudent use of antibiotics, application of biosecurity measures and dietary interventions to mitigate the development and spread of resistance (107).

### Gentamycin

Gentamycin is a broad-spectrum bactericidal aminoglycoside that inhibits protein synthesis and used widely against Gram-negative and some Gram-positive bacteria (108). In Canada and USA gentamycin is approved in day-old chicks at the hatchery to prevent early mortalities (109), while controlling *E. coli, Salmonella*, and *Pseudomonas* infections (98). A high prevalence of gentamycin resistant *E. coli* have been reported in Ontario in chicks of less than 10 days old. The increase in the use and resistance of gentamycin warrants further surveillance to determine whether production stage, season, age, and year are predictors of resistance to this antibiotic (110). The increased use of gentamycin at the hatchery to control *E. coli* (omphalitis) infection has been associated with its higher resistance level during the brooding stage (109). Further studies are required to ascertain AMR at the hatchery and brooding stage. Comparative phenotypic and genotypic analyses of resistant and susceptible *E. coli* strains in these rearing environments would provide insight into a genetic adaptation that confers resistance. A study on AMR phenotype and genotype in *E. coli* isolates from lesions of colibacillosis chicken has highlighted the difference in gene expression and the presence of specific resistance genes in resistant strains (105). Gentamycin resistance is commonly due to aminoglycoside-modifying enzymes acetyltransferases *(aac)* and O-phosphotransferases *(aph).* The aminoglycosides resistance *aph(2″), aad(A),* and *aac(6″)* genes are widespread in *E. coli* and prevalent in poultry and their environment (111). These genes are frequently found on plasmids, integrons, and transposons that contribute to their dissemination (112).

The lincomycin-Spectinomycin combination is used against respiratory and gastrointestinal infections in poultry. This practice has been linked to an increased gentamycin resistance mediated by *aac(3)*, *aph(2″),* and *aad(A)* on plasmids, indicating potential horizontal gene transfer (113–115). Genomic investigation of gentamycin resistance in *E. coli* isolates from human and chicken sources in Canada between 2014 and 2017 revealed that the use of lincomycin-spectinomycin on poultry farms might be co-selecting for gentamycin-resistant plasmid in *E. coli* in broilers (116). Due to the development and increased AMR, the implementation of guidelines to restrict the use of gentamycin and other antibiotics in poultry is required. Alternative options include dietary supplementation with feed additives, vaccines, and biosecurity measures (117).

The 16S rRNA genes coding for the 16S ribosomal RNA are used in bacterial phylogeny building. The methylations of the 16S RNA due to methylases, including ArmA, RmtA/B/C/D/E/F/G/H, and NmpA, have been shown to induce, high level resistance to amikacin, tobramycin, gentamicin, and netilmicin. Aminoglycoside resistance due to ArmA and RmtB has been reported in poultry *E. coli* isolates (118).

### Beta-lactams

Amoxicillin has been used in some countries such as Australia against infectious coryza (*Avibacterium paragallinarum*); fowl cholera (*Pasteurella multocida*), *Ornithobacterium rhinotracheale* and *E. coli* (119). Despite a reported low level of AMR, beta-lactam resistant *E. coli* harboring the beta-lactamase *bla*_TEM-1B_ gene have been detected in Australian layer hens (120). Extended spectrum beta-lactamase (ESBL) genes including *CTX-M* and *SHV* alleles have been reported in poultry *E. coli* isolates in Europe, United Kingdom, China and Nigeria (118). With the emergence of multidrug-resistant *E. coli* strains in poultry, a search for vaccines against APEC has been conducted.

### Vaccines

The current strategy against APEC is based on good sanitation and hygiene, which seem to be adequate but insufficient to control colibacillosis in commercial setups. Given their antigenic variability, finding efficient vaccines to prevent APEC infections has proved challenging (121). The vaccine is deemed successful in protecting against APEC infections with homologous strains but less effective against heterologous strains (63). Various virulence-associated genes have recently been evaluated as vaccine candidates to prevent colibacillosis in layer hen (122–124). The use of a live attenuated *Salmonella* delivery system with a recombinant construct that harbors genes encoding different virulence factors of APEC induced a robust immune response to prevent colibacillosis (84, 125, 126). Despite that reports showed the efficacy of the *ΔaroA* vaccine and its potential to reduce the virulence of APEC, the large molecular diversity of APEC strains that have hampered its effectiveness (127, 128). A combination of several virulence-associated genes (VAG) is required to confer pathogenicity in APEC, and no single or sets of certain VAG are associated with APEC, hence making it difficult to designs vaccines that would target all APEC strains. With these challenges, control measures and strategies, including suitable housing infrastructure and dietary intervention, are deemed helpful in bolstering the immunity and survival capability in pullets and layers.

### Non-antibiotic dietary interventions

#### Probiotics: bacteria and yeast

The use of probiotics as alternatives to antibiotics and immune modulators is gaining tremendous interest in the poultry industry. According to the FAO/WHO, probiotics are defined as “*live microorganisms which when administered in adequate amounts confer a health benefit on the host*” (129). Probiotic bacteria used in animal production are generally Gram-positive and belong to *Bacillus* spp. (*B. cereus* var. *toyoi*, *B. licheniformis*, *B. subtilis*), *Enterococcus* spp. (*E. faecium*), *Lactobacillus* spp. (*L. acidophilus*, *L. casei*, *L. farciminis*, *L. plantarum*, *L. reuteri*, *L. rhamnosus*), *Pediococcus* spp. (*P. acidilactici*), and *Streptococcus* spp., (*S. infantarius*). *Lactobacillus* spp., *Enterococcus* spp., *Pediococcus* spp., *Bacillus* spp. *Saccharomyces* are the most common probiotics used in the poultry industry to improve product safety, feed efficiency, bird health, performance, and immunity (116, 130). *Bifidobacterium* spp. probiotics are widely used in combination with *Lactobacillus* spp. and other combination products. *Bifidobacterium* directly increases secretions of IgA in the intestinal tracts and stimulates phagocytes and pancreatic elastase productions through secretion of the serine protease inhibitor serine-production. This pro-inflammatory response mechanism suggests *Bifidobacterium* spp. and serine productions participate in gut microbiota homeostasis (131). Furthermore, *Bifidobacterium* spp. produces lactate and acetate, which are later used as gut fermenters to produce butyrate and propionate that aid in improving gut health (132). A fiber-rich diet could lead to high acetate levels generated by beneficial gut bacteria such as *Bifidobacterium* to provide significant protective effects (133).

In laying hens, *B. subtilis* supplemented diets (0.5 g/kg) of laying hens promote growth performance and balance the gut microbiota by inhibiting *E. coli* and *Clostridium perfringens* colonization Table 3 (134). More studies have shown that *Bacillus*-based probiotics could decrease *E. coli* in chickens, by altering microbiota composition and community structure (121, 135, 136). Lei et al. (135), and Hetab et al. (136) demonstrated that a *B. subtilis*-supplemented diet bolsters the immunity of chicks and enhances the performance and egg quality in layers. *Lactobacillus* spp. is produced during the sugar metabolism process; lactate inhibits the growth of pathogenic bacteria by reducing intestinal pH or directly interfering with normal bacteria metabolism. Certain *Lactobacillus* spp. species produce bacteriocin compounds, which are bactericidal to pathogenic microbes such as APEC, *Salmonella* spp., and *Clostridium* spp. (137). However, the type of *Lactobacillus* probiotics supplemented in the diet, for instance, *L. acidophilus* probiotic-fed hens at 18 weeks, 5 months, and 7 months, yielded an extremely low number of coliforms, *E. coli*, *Clostridium* spp., and *Staphylococci* spp. in ceca and ileal contents (137, 138). Although the total anaerobes population was less affected by the probiotics.

**Table 3:** Dietary inclusions of functional feed additives for laying hens and their effects on some gut microbiota and *E. coli.*

Feed additives	Age/Strain	Dosage/form	Duration (wks)	Outcome	Year of publication	Ref.
XOS (Pre)	28 wk. old White Lohmann hens	0 to 0.05%/diet	8	**↑** Villus height, villus-to-crypt depth ratio, SCFA, TNF-alpha, IL-2 **↑** *Bifidobacterium spp*	2017	(175)
MOS (Pre)	68 wks old Hy-Line White	0 to 2 g/kg/diet	11	**↑** Ileal nutrition digestibility **↓** *Salmonella, E. coli*	2016	(220)
MOS (Pre)	55 wks old Hy-Line White	0.05–0.2%/diet	11	**↑** *Lactobacillus* **↓** *Salmonella* **—** *E. coli*	2015	(157)
*B. subtilis* (Pro)	64 wks old Lohmann LSL-Classic hens	0.5 g/kg/diet	9	**↑** *Lactobacillus, Bifidobacterium* **↓** *Clostridium, Coliform (E. coli)*	2013	(134)
*B. subtilis* (Pro) Inulin (Pre) *B. subtilis* and Inulin (Syn)	64 wks old Lohmann LSL-Classic hens	1 g/kg *B. subtilis*/diet 1 g/kg inulin/diet	12	**↑** *Lactobacillus, Bifidobacterium* **↑** Villus height, villus-to-crypt depth ratio **↓** *Clostridium, Coliform (E. coli)*	2013	(134)
THY + CIN (EOs)	50 wks Hy-line brown hens	100 mg/kg/diet	6	**↑** *Bifidobactericiae, Lactobacillus* **↑** Villus height, villus-to-crypt depth ratio	2022	(172)
Flaxseed oil/Marine algae	20 wks old Lohmann LSL-Classic hens	0.20 & 0.60%/diet	8	**↑** *Firmicutes, Bacteoidetes Ruminococcaceae*	2020	(183)
Live Yeast (Pro)	36 wks old Hy-line layers	0.4 and 0.8%/diet	4	**↑** *Lactobacillus* **↓** *E. coli, Klebsiella* spp.*, Staphylococcus,* **↓** *Campylobacter* spp. and *C. perfringens*	2010	(142)
Yeast culture (Pro)	54 wks Hy-Line Brown	2.0 g/kg/diet	8	**↑** *Lactobacillus, Bacilli* **↑** *E. coli*	2021	(144)
Inulin (Pre)	50-wk Brown Nick	2.0%/diet	4	**↑** *Lactobacillus, Bifidobacteria* **↓** *Colform/E. coli*	2010	(152)
Inulin (Pre)	30- Hy-Line Brown	15 g/kg/diet	8	**↑** *Bacteroidetes, Firmicute*, SCFA	2020	(154)

Pre, prebiotics; Pro, Probiotics; EOs, Essential oils; Syn: synbiotics; LSL, Lohmann Selected Leghorn; SCFA, shorty chain fatty acid; TNF, tumor necrosis factor; wks, weeks; increase (↑); decrease (↓); No change (—).

*Saccharomyces cerevisiae* is among the most yeast probiotics investigated for its potential beneficial effects in layers (139). Feeding pullets with diets supplemented with live yeast probiotic improves gut health and bolster immunity by increasing hematological profiles for total erythrocytes and leukocyte cell counts, marked by an increase in lymphocyte percentage (140). It has been found that the live yeast probiotics stimulated the immune system of pullets by increasing lymphocyte proliferation which helps increasing infection resistance in pullets (141). Dietary supplementation with live yeast probiotics (2.0 g/kg) for 8 weeks in laying hens has been shown to modulate the bird intestinal microflora by inhibiting the colonization of the GIT by enteric pathogens (*E. coli*, *Salmonella*, *Campylobacter jejuni*, and *C. perfringens*) (142), this promotes the immune response of the birds and enhances performance (143). Feeding poultry with live yeast supplements improves the ileum’s microbial population by increasing lactic acid bacteria and reducing *E. coli*, as shown in Table 3 (144).

Probiotics mechanism of actions include improving the microbial environment of a bird’s intestinal tract by displacing pathogenic microbes (145), competing for receptors on the gut mucosa necessary for attachment proliferation and colonization by beneficial microorganisms, which prevents the establishment of pathogens in the gut, and inhibiting growth of competitors by the production of primary and secondary (antimicrobial and immune modulation) metabolites (145). Dietary probiotics stimulate both cell-and humoral-mediated immunity through enhancing production cytokines/interferon, increased macrophages, lymphocytes, and natural killer cells (NK) activity, upregulated oxidative burst in heterophils, and increased immunoglobulins production/functions. In the gut, probiotics increase the number of lymphocytes and gut-associated lymphoid tissue in lamina propria and intra-epithelial lymphocytes, which could inhibit the growth of pathogenic organisms, especially during the transition from pullets to hens (38). However, stressors in poultry reduce the growth and functions of immune organs such as the bursa of Fabricius, thymus, and spleen (146), impairing immune responses and disease resistance (109).

#### Prebiotics

Prebiotics are defined as non-digestible ingredients that selectively promote the growth of beneficial bacteria in GIT, enhancing gut health and potentially improving the host’s health (111). Prebiotic compounds encompass numerous indigestible oligosaccharides such as fructooligosaccharides (FOS) products (oligofructose, inulin), trans-galactooligosaccharides (GOS), glycooligocharides, maltooligosaccharides, xylo-oligosaccharides (XOS), yeast cell wall (mannan oligosaccharides), glucooliogasaccharides and glycooligosacharids (115). The mechanism of actions of prebiotics in improving poultry health include pathogen’s inhibition by binding to the intestinal epithelium and generation of short-chain fatty acids (SCFA). The production of SCFA in the host intestine by prebiotic fermentation provides energy for epithelial cells, which reduces luminal pH and acts as the signaling molecule influencing the immune cells’ activities (147).

Dietary prebiotics have been shown to enhance the immunity in poultry. A significant upregulation of IL-10 (interleukin-10) was demonstrated in non-challenged Lohmann pullets fed yeast-derived carbohydrates for two weeks after post-hatch (148). This interleukin (IL-10) exerts its effects by modifying the immune response of different immune cells such as T, B, APC, and natural killer cells. Dietary beta-glucan has been shown to induce the expression of immune regulatory cytokines such as IL-10, transforming the growth factor-β1 and IL-2 in the bone marrow and spleen (149). Prebiotics (beta-glucan, FOS, inulin, and MOS) interact with pathogen-associated molecular pattern (PAMP), activating immune cells including macrophages, dendritic cells, and neutrophils, resulting in an increased the cytokines and other immune mediators’ production (150, 151). These activities of prebiotics can change the expression of genes associated with immune responses, enhancing birds’ ability to combat infections.

Dietary supplementation with inulin at 1, 1.5, and 2% has been shown to decrease the egg cholesterol contents in 50 to 54 weeks brown Nick laying hens (152). Furthermore, diet supplementation with inulin for 8 weeks in 30-weeks old Hy-Line brown laying hen induced an improved egg production and serum antioxidant activities in the group of 10, 15, and 20 g/kg (153), the improved egg production was due to an increased nutrient digestibility and the selective modulation of cecal microbial communities Table 3 (154). Prebiotics mannan oligosaccharides (MOS) are derived from yeast (*S. cerevisiae*); the mechanism of MOS in prevention against Gram-negative bacteria such as *E. coli* and *Salmonella,* is through to be through binding to type 1 fimbria (155). If the pathogen fails to bind to the receptor, they are flushed out constantly via excreta. *In vitro* studies have shown that additional MOS inhibits the attachment of enteropathogenic *E. coli* to the gut mucosa and removes the attached *E. coli* from the mucosa (156). In addition, FOS or MOS have been reported to decrease feed conversion, increase body weight gain, and egg production (157).

Feed enzymes (FEs) routinely used in poultry nutrition to aid in the hydrolysis of specific chemical bonds in feedstuffs, elimination of nutrients encapsulating effects of cell wall polysaccharides, resulting in the availability for absorption, and breakdown of feed antinutritional factors (158). Dietary inclusion with FEs in viscous feedstuffs could promoted growth performance, improved digestibility, and decreased feed cost. Short-chain xylo-oligosaccharides derived from *in vitro* hydrolysis of wheat bran by endoxylanases then fed to broilers resulted in an increased population of *Bifidobacteria* in the caeca and enhanced feed conversion ratio (159). Beta-mannanase modulates the feed-induced immune response and gastrointestinal ecology by hydrolyzing native beta-mannan to smaller fragments with reduced ability to stimulate the innate as demonstrated by the mucosal permeability, modulation of oxidative stress and concentration of acute phase protein and immunoglobulins in poultry (160).

### Symbiotics

Prebiotics and probiotics combinations are known as symbiotics, which synergistically enhance gut health and immune functions (161). Studies have demonstrated the potential benefits of symbiotics on the intestinal microbiota and immune functions of pullets and laying hens (Table 3). The symbiotic mechanism of actions seems to be like prebiotics and probiotics (162). It is hypothesized that prebiotics and probiotics act independently in the gut. Prebiotics (indigestible oligosaccharides) are fermented in the gut, whereas probiotics (beneficial live microorganisms) colonize the gut. Moreover, other mechanisms of symbiotics might be to promote both pro-and anti-inflammatory responses in the host by regulating gut microbiota, increasing the population of beneficial bacteria, which up-regulates immune metabolic pathways and affects T cell maturation (163).

*Lactobacillus salivarius* and GOS have been shown to stimulate the gut-associated lymphocyte tissue (GALT) by T and B cells of broiler chicken (164). Al-Fataftah et al. (134) study has revealed that dietary inclusion of *B. subtilis* (1 g/kg) and inulin (1 g/kg) supplementation in (symbiotic) in laying hens improves eggshell quality, gut morphology, and growth of beneficial bacteria, as shown in Table 3. *Bifidobacterium* spp. and *Lactobacillus* spp. are some gut microbiotas that utilize inulin to ferment indigestible carbohydrates, produce SCFAs, stimulate immunoglobulin production, and help in competitive exclusions of pathogens by promoting the growth of beneficial bacteria (165). Dietary supplementation with inulin significantly increased *Bifidobacterium* spp., lowered pH, and reduced *E. coli*/coliform count in caeca of laying hens (152). Gut microbiota is pH sensitive; hence, its reduction is accompanied by changes in bacteria composition, such as inhibiting the proliferation of pathogenic *E. coli* and *Salmonella* spp. (165). Dietary supplementation with *Lactobacillus* spp. and *S. cerevisiae* derivatives increased *Bifidobacterium* and *Lactobacillus* populations in broiler’s gut while decreasing population of potentially pathogenic microbes such as *Clostridium* spp. and APEC (166). A study by Wareth (167) on Thymol and the symbiotics (*Enterococcus faecium* and fructooligosaccharides), alone or in combination in diet, improved egg production, egg mass, and feed conversion ratio from 24 to 36 weeks of age.

### Phytogenics

Phytogenic feed additives, also known as botanicals or phytobiotics/phytochemicals are derived from plants and utilized in animal nutrition to promote growth and health (168). They include herbs, spices, other plants, or their extracts, such as essential oils (EOs). Phytobiotic compounds can be grouped into five categories: phenolic, alkaloids, nitrogen-containing agents, phytosterols, and carotenoids, each acting differently in improving the poultry immunity and health (169). Their actions include inhibiting microorganisms, disrupting metabolic processes, modulating signal transduction pathways, and immunomodulation via gene expressions (65). Phytobiotics could decrease intestinal inflammation and improve barrier functions by inhibiting toll-like receptors and subsequent activate NF-kb, the xenobiotics detoxifying system and the nuclear factor erythroid-related factor 2 (Nrf2) pathway as well as inhibit pathogenic bacteria. This enhancement of intestinal functions prevents the translocation of pathogens and harmful substances into the bloodstream by induction of systemic inflammation via excess secretion of cytokine and glucocorticoids (169). Dietary supplementation with cranberry pomace and extract resulted in upregulation of anti-inflammatory IL-10 in broilers (170). This interleukin (cytokine) also is involved in improving immune response including immunoglobulin production, increase NK cells and CD8+ T cells cytotoxic activities, as well as the thymocytes proliferation (171). These could help explain the decreased gut enteric bacteria including *E. coli* counts following berry products feeding as reported by Das et al. (170).

Previous studies on dietary EOs (thyme, rosemary, garlic, and sage) in laying hens (Table 3) reported improved performance, enhanced immune response, and promoted the growth of beneficial bacteria (172–174). Dietary inclusions of EOs mixtures at 24 mg/kg improved egg production, feed efficiency, hens while reducing the percentage of cracked eggs (175). The effectiveness of EOs’ depends on chemical structure, pH, concentration of bioactive compounds, and microbial population and strains present (176). EOs are considered more effective against Gram-negative than Gram-positive bacteria because their outer membrane is enclosed by a cell wall that restricts entry of hydrophobic compounds by lipopolysaccharides cell structure. Most EOs promote the growth of beneficial bacteria in the gut and moderate pathogenic bacteria in poultry, for example, *Clostridium perfringens* and *E. coli* (177). EOs from *Thymus* and *Origanum-species* have a high antimicrobial activity associated with their phenolic compounds such as thymol and carvacrol. The combination of EOs eugenol/carvacrol and eugenol/thymol showed a synergistic effect, whereby thymols and carvacrol disintegrated the outer membrane of *E. coli*, *making* it easier for eugenol to access bacterial cytoplasm to inhibit their growth (31). It has been reported that dietary encapsulated cinnamaldehyde could influence AMR virulence of *E. coli* in broiler however, more research would be needed to establish the involved mechanisms (178).

### Fatty acids

Fatty acids are essential components of diets which are crucial in energy provision, cell structure, and regulation of body functions. Fats are the building blocks for fatty acids and are categorized based on their chemical structure and properties: saturated fatty acids have no double-bonds, monosaturated fatty acids (MUFA) have one double-bonds between carbon atoms, and polyunsaturated fatty acids (PUFA) have multiple bonds between carbon atoms (179). Dietary inclusion of n-3 PUFA has gained interest due to its potential health benefits by enhancing immunity in broiler chickens. Higher antibody titer against Newcastle disease virus were observed in 60-week n-3 PUFA fed-hen than the control corn-based diet (180). Similarly, feeding pullets breeders with n-3 PUFA (DHA and ALA) elevated embryogenic utilization of docosahexaenoic acid (DHA) and antibody titers against Newcastle and infectious bronchitis disease during the post-vaccination period (181, 182). However, further studies are needed to ascertain whether the antibody titer depends on the dosage or source of n-3 PUFA.

Dietary flaxseed oil (0.20 and 0.60%) in laying hens has been shown to promote the growth of beneficial bacteria, as shown in Table 3 (183). Feeding 46-weeks Lohmann layers with algae oil increased the concentration of DHA with no effects on egg production and eggshell quality (184). However, more studies are required to understand the correlation between gut microbiota, egg production, and enrichment in DHA. Effects of increasing concentration of n-3 PUFA (0.2, 0.4, 0.6, and 0.8%) from either flaxseeds oil or preformed docosahexaenoic acid on fatty acid composition and immune response of 20-week of age LSL lite laying hen, challenged with *E coli*-derived LPS (8 mg/kg via intravenous injection) were investigated (185). These authors showed that LPS increased the mRNA expression of proinflammatory cytokine IFN-*γ* and receptor TLR-4 (185). Similarly, n-3 FA was found to influence LSL lite pullets’ body weight, development of lymphoid organs, and some plasma metabolites (67). Wang et al. (186), found that dietary supplementation of n-3 PUFA alters lymphocyte subset proportion and immune tissue development of chick by promoting the growth of the spleen, thymus, and bursa of Fabricius before 4 weeks. The bursa withers or becomes damaged during 4 to 8 weeks of age. Despite variations in formulation and dosage, these findings suggest a strong link between n-3 PUFA acid and immune development, indicating its potential immunomodulatory feature as feed additives in pullets and laying hens. However, long-term effects induced by dietary n-3 PUFA on chicken immunity, resistance to relevant infectious challenges, and chicken performance remain the subject of research.

## Challenges of nutritional interventions and future direction

Heat treatment during feed pelleting at the feed mill does not totally kill bacteria such as *E. coli* and *Salmonella*. The bacteria might be present in the final feed, representing a potential risk of transmission via feed (187, 188). A fiber-rich diet results in higher production of butyrate by the intestinal microbiota, which promotes the expression of the host’s globotriaosylceramide, an enterocyte receptor for the Shiga toxins (189). Therefore, promoting the growth of acetate-generating bacteria by dietary interventions could be a valuable strategy in mitigating the risk and severity of toxin-mediated diseases.

Dietary supplements have shown inconsistency in improving the performance and health of pullets and hen’s response which could be influenced by of dosage, age, sex, and breeds. As discussed earlier, a broad array of possibilities contributes to variation in feed supplement efficacy. Feed additives have pleiotropic actions including competitive exclusion, promoting growth of beneficial bacterial and producing antimicrobial metabolites. More detailed information is needed to help understand how combining nutritional intervention with optimal age-specific dosage, improved biosecurity, vaccination, and other health management practices in pullets and laying hens can provide a synergistic effect, reducing the overreliance on single strategies, and this might help in improving the health of laying hens and reducing APEC associated infections.

The restriction of AMU in poultry production and the search of other alternative strategies could have a complex impact on AMR in APEC infections (190). Alternatives, such as prebiotics, probiotics, and phytogenic, among other feed additives, cannot completely protect against pathogens, potentially resulting in increased use of therapeutic antibiotics. Thus, controlling measures to mitigate AMR must include enhanced biosecurity measures, responsible AMU, and further research in dietary interventions is required (191). The findings from breeder grandparent flocks have established them to be a possible source and reservoir for APEC strains (192). These strains can harbor and transmit *E. coli* vertically down the breeding pyramid to their progeny (192). A thorough and comparative analysis of the *E. coli* genome is necessary to ascertain it. Additionally, a longitudinal and molecular diversity study of the *E. coli* genome is required to evaluate and understand how the rearing environment and dietary supplements contribute to shaping adaptation and transmission of colibacillosis-associated *E. coli* in layers production system (192).

## Conclusion

Colibacillosis induced by APEC affects pullets and hens of all age groups. The risks of infection with APEC and its evolution increase with the increased environmental pressure (housing, density, bird immunity, among other stressors). Hatchery hygiene, a good housing environment, and early feeding to boost immunity are important in controlling colibacillosis. Dietary supplementation with functional feed additives benefits pullets and layer hens. It results in improved egg production, bolsters immunity, promotes a healthy gut, and protects against colibacillosis. However, there is a need to standardize the dosage, breed, time, health status, and age of birds while administering these functional feed additives as management strategies for colibacillosis. Avian colibacillosis in chicks causes high mortality in the first week after hatching; it may be logical to introduce feed additives (probiotics, prebiotics, symbiotics and phytobiotics) early in the life of chicks, as this would help bolster immunity and promote a healthy gut. Improved gut health aided by gut microbiota plays a significant role in disease dynamics, host-pathogen interaction, host-commensal interaction, and commensal pathogen interaction in the GIT of the birds under dietary supplemented with functional feed additives. Furthermore, the development of sequencing technologies and bioinformatics pipelines would also make it possible to delineate some of the complex associations between the wide range of feed additives and poultry GIT microbial response to dietary feed additive as well as to identify drug and/or vaccine target against APEC.
